# World Allergy Organization-McMaster University Guidelines for Allergic Disease Prevention (GLAD-P): Prebiotics

**DOI:** 10.1186/s40413-016-0102-7

**Published:** 2016-03-01

**Authors:** Carlos A. Cuello-Garcia, Alessandro Fiocchi, Ruby Pawankar, Juan José Yepes-Nuñez, Gian Paolo Morgano, Yuan Zhang, Kangmo Ahn, Suleiman Al-Hammadi, Arnav Agarwal, Shreyas Gandhi, Kirsten Beyer, Wesley Burks, Giorgio W. Canonica, Motohiro Ebisawa, Rose Kamenwa, Bee Wah Lee, Haiqi Li, Susan Prescott, John J. Riva, Lanny Rosenwasser, Hugh Sampson, Michael Spigler, Luigi Terracciano, Andrea Vereda, Susan Waserman, Holger J. Schünemann, Jan L. Brożek

**Affiliations:** Department of Clinical Epidemiology & Biostatistics, McMaster University, Hamilton, ON Canada; Tecnologico de Monterrey School of Medicine, Monterrey, Mexico; Pediatric Hospital Bambino Gesù, Vatican City, Italy; Department of Pediatrics, Nippon Medical School, Tokyo, Japan; University of Antioquia, School of Medicine, Medellín, Colombia; Department of Pediatrics, Samsung Medical Center, Sungkyunkwan University School of Medicine, Seoul, Korea; Department of Pediatrics, College of Medicine and Health Sciences, United Arab Emirates University, Al-Ain, United Arab Emirates; Faculty of Medicine, University of Toronto, Toronto, ON Canada; Charité Klinik für Pädiatrie, Berlin, Germany; Department of Pediatrics, University of North Carolina, Chapel Hill, NC USA; University of Genoa, IRCCS AOU San Martino-IST, Genoa, Italy; Department of Allergy, Clinical Research Center for Allergology and Rheumatology, Sagamihara National Hospital, Kanagawa, Japan; Department of Pediatrics and Child Health, Aga Khan University Hospital, Nairobi, Kenya; Department of Paediatrics, Yong Loo Lin School of Medicine, National University of Singapore, Singapore, Republic of Singapore; Department of Primary Child Care, Children’s Hospital, Chongqing Medical University, Chongqing, China; Department of Immunology, Perth Children’s Hospital, Telethon KIDS Institute, School of Paediatrics and Child Health, University of Western Australia, Crawley, Australia; Department of Family Medicine, McMaster University, Hamilton, ON Canada; Allergy-Immunology Division, Children’s Mercy Hospital & University of Missouri – Kansas City School of Medicine, Kansas City, MO USA; Jaffe Food Allergy Institute, Icahn School of Medicine at Mount Sinai, New York, NY USA; Food Allergy Research & Education (FARE), McLean, VA USA; Department of Child and Maternal Medicine, University of Milan Medical School at the Melloni Hospital, Milan, Italy; Allergology Department, Hospital Infantil Universitario Niño Jesus, Madrid, Spain; Department of Medicine, McMaster University, Hamilton, ON Canada

## Abstract

**Background:**

The prevalence of allergic diseases in infants, whose parents and siblings do not have allergy, is approximately 10 % and reaches 20–30 % in those with an allergic first-degree relative. Intestinal microbiota may modulate immunologic and inflammatory systemic responses and, thus, influence development of sensitization and allergy. Prebiotics – non-digestible oligosaccharides that stimulate growth of probiotic bacteria – have been reported to modulate immune responses and their supplementation has been proposed as a preventive intervention.

**Objective:**

The World Allergy Organization (WAO) convened a guideline panel to develop evidence-based recommendations about the use of prebiotics in the prevention of allergy.

**Methods:**

The WAO guideline panel identified the most relevant clinical questions about the use of prebiotics for the prevention of allergy. We performed a systematic review of randomized controlled trials of prebiotics, and reviewed the evidence about patient values and preferences, and resource requirements (up to January 2015, with an update on July 29, 2015). We followed the Grading of Recommendations Assessment, Development and Evaluation (GRADE) approach to develop recommendations.

**Results:**

Based on GRADE evidence to decision frameworks, the WAO guideline panel suggests using prebiotic supplementation in not-exclusively breastfed infants and not using prebiotic supplementation in exclusively breastfed infants. Both recommendations are conditional and based on very low certainty of the evidence. We found no experimental or observational study of prebiotic supplementation in pregnant women or in breastfeeding mothers. Thus, the WAO guideline panel chose not to provide a recommendation about prebiotic supplementation in pregnancy or during breastfeeding, at this time.

**Conclusions:**

WAO recommendations about prebiotic supplementation for the prevention of allergy are intended to support parents, clinicians and other health care professionals in their decisions whether or not to use prebiotics for the purpose of preventing allergies in healthy, term infants.

**Electronic supplementary material:**

The online version of this article (doi:10.1186/s40413-016-0102-7) contains supplementary material, which is available to authorized users.

## Executive summary

The purpose of this document is to provide evidence-based recommendations about the use of prebiotic supplements for the primary prevention of allergies.

Allergic diseases have a prevalence of 10 % in infants without an allergic parent or sibling, and 20 – 30 % in those with allergy in their relatives. It has been suggested that intestinal microbiota may modulate immunologic and inflammatory systemic responses and, thus, influence the development of sensitization and allergy. Prebiotics are non-digestible food components that have been advocated as preventive interventions for allergic diseases by promoting a balanced growth of the intestinal microbiota.

### Methodology

The methods used to develop clinical recommendations in this document follow the GRADE approach [1, 2]. The Guideline panel included clinicians and researchers in the field of allergy (allergists), pediatricians, primary care physicians, and methodologists. Potential conflicts of interests were managed as suggested by the World Health Organization.

The guideline panel developed and graded the recommendations and assessed the quality of the supporting evidence following the GRADE approach. The quality of evidence (also called *confidence in the available estimates of health effects* or *certainty in the evidence*) is categorized as: high, moderate, low or very low based on consideration of risk of bias, indirectness of evidence, inconsistency and imprecision of effect estimates. Low and very low quality evidence indicates that the estimated effects of interventions are very uncertain.

### Interpretation of strong and conditional recommendations

The strength of a recommendation is expressed as either strong (“guideline panel recommends…”), or conditional (“guideline panel suggests…”) and has the following interpretation:

#### Strong recommendation

- ▪ For patients: most individuals in this situation would want the recommended course of action, and only a small proportion would not.
- ▪ For clinicians: most individuals should receive the intervention. Adherence to this recommendation according to the guideline could be used as a quality criterion or performance indicator. Formal decision aids are not likely to be needed to help individuals make decisions consistent with their values and preferences.
- ▪ For policy makers: the recommendation can be adopted as policy in most situations.

#### Conditional recommendation

- ▪ For patients: the majority of individuals in this situation would want the suggested course of action, but many would not.
- ▪ For clinicians: recognize that different choices will be appropriate for individual patients and that you must help each patient arrive at a management decision consistent with his or her values and preferences. Decision aids may be useful in helping individuals to make decisions consistent with their values and preferences.
- ▪ For policy makers: policy-making will require substantial debate and involvement of various stakeholders.

### How to use these guidelines

The GLAD-P guidelines about the use of prebiotics provide the basis for rational, informed decisions for clinicians, parents and other decision makers. Clinicians, parents and other caregivers, third-party payers, institutional review committees, other stakeholders, or the courts should never view these recommendations as dictates. No recommendation can take into account all of the often-compelling unique individual circumstances but provides guidance. However, no one charged with evaluating health care professional’s actions should attempt to apply the recommendations from these guidelines by rote or in a blanket fashion.

Note: statements about the underlying values and preferences as well as qualifying remarks accompanying each recommendation as well as the evidence to decision frameworks are its integral parts and serve to facilitate more accurate interpretation; they should never be omitted when quoting or translating recommendations from these guidelines.

### Recommendations

**Question 1. Should prebiotics be used in pregnant women?**

The WAO guideline panel chose to provide no recommendation at this time owing to lack of experimental and observational studies in which prebiotic supplements would be used in pregnant women.

**Question 2. Should prebiotics be used in breastfeeding mothers?**

The WAO guideline panel chose to provide no recommendation at this time owing to lack of experimental and observational studies in which prebiotic supplements would be used in breastfeeding mothers.

**Question 3. Should prebiotics be used in healthy infants?**

**Recommendation:** The WAO guideline panel suggests prebiotic supplementation in not-exclusively breastfed infants, both at high and at low risk for developing allergy (conditional recommendation, very low certainty of evidence).

The WAO guideline panel suggests that clinicians and parents do *not* use prebiotic supplementation in exclusively breastfed infants (conditional recommendation, very low certainty of the evidence).

#### Values and preferences

The recommendation to use prebiotics in not-exclusively breastfed infants places a relatively higher value on possible prevention of allergies and a relatively lower value on additional cost of prebiotic supplementation. The recommendation not to use prebiotics in exclusively breastfed infants places a relatively higher value on avoiding additional cost and burden of implementation of such supplementation and a relatively lower value on uncertain effect on prevention of allergies (currently there are also no studies of health effects of prebiotics in exclusively breastfed infants and the panel found it not justified to extrapolate from the effects observed in non-exclusively breastfed infants).

#### Explanations and other considerations

These recommendations should not lead to any change in the practice or promotion of breastfeeding in infants. Formulas supplemented with prebiotics should not be considered a substitute for breast milk. Sole availability of such formulas should not influence the decision to breastfeed and/or the duration of breastfeeding. These recommendations apply to otherwise healthy term infants in whom prebiotics would be used with the goal of preventing allergies.

## Scope and purpose

The purpose of this document is to provide guidance on the use of prebiotics for the primary prevention of allergies. The target audience of these guidelines are general practitioners, paediatricians, allergists, pulmonologists and dermatologists. General internists, and other health care professionals and health policy makers may also benefit from these guidelines. Policy makers interested in these guidelines include those involved in developing local, national or international plans with the goal to reduce incidence of allergy and resource direct and indirect costs related to allergic diseases [3]. This document may also serve as the basis for development and implementation of locally adapted guidelines.

## Background

Allergic diseases are considered a worldwide burden in different populations. It is considered that approximately 20 % of the population will have an allergic disease at some point in their lives [4]. The prevalence of allergic diseases in infants depends on the allergic status of their parents; for example, the prevalence is 20 – 30 % in those with an atopic background in their first-degree relatives, while in those without an allergic parent or sibling the risk decreases to about 10 % [5].

Recent studies have provided researchers more reasons to move their attention to the intestinal microbiota as one of the main culprits for the rising incidence of allergic disorders; its influence on sensitization and its ability to modulate immunologic and inflammatory systemic responses are the main reasons for this suspicion; [6] in consequence, the “hygiene” or “microflora” hypothesis has been proposed to explain these phenomena [7].

Prebiotics have been defined as “non-digestible food components that beneficially affect the host by selectively stimulating the growth and/or activity of one or a limited number of bacteria in the colon and thereby improving host health” and later redefined as”a selectively fermented ingredient that allows specific changes, both in the composition and/or activity in the gastrointestinal microbiota that confers benefits” [6, 8, 9].

The Guidelines for Atopic Disease Prevention (GLAD-P) is a joint effort of the World Allergy Organization (WAO) and the Department of Clinical Epidemiology & Biostatistics at McMaster University to evaluate the current evidence on the preventive effect of probiotics, prebiotics, and vitamin D on allergic diseases and related clinically important outcomes. This document provides recommendations and the rationale for use of prebiotics.

For clarity of communication we used the following definitions throughout the document:

- ***Prebiotics:*** “a selectively fermented ingredient that allows specific changes, both in the composition and/or activity in the gastrointestinal microbiota, thus conferring benefit(s) upon host health.” [6, 8, 9]. Prebiotics most commonly used as components of infant feeds are non-digestible carbohydrates; mostly fructans like inulin, oligofructose, and fructooligosaccharides (FOS); other prebiotics in use include isomaltooligosaccharides, soybean oligosaccharides, gentiooligosaccharides, galactooligosaccharides (GOS), and xylooligosaccharides. These are substances primarily derived from plants and commonly found in foods like garlic, onion and artichoke, among others. To consider a substance a prebiotic, this must: a) resist gastric acidity, lysis by enzymes, and absorption in the gastrointestinal tract; b) be fermented by intestinal microbiota; and c) selectively stimulate the growth and/or activity of intestinal bacteria potentially associated with health and well-being [8, 9].
- ***High risk for allergy in a child***: biological parent or sibling with existing or history of allergic rhinitis, asthma, eczema, or food allergy [10].

## Methods

### Panel composition and meetings

We followed the procedures and methodology using the GIN-McMaster Guideline Development Checklist (http://cebgrade.mcmaster.ca/guidecheck.html) [2] to assemble a team of experts including allergists, paediatricians, family physicians and representatives of the general public. The guideline panel also included methodologists who helped prepare systematic reviews and evidence summaries following the GRADE approach.

A face-to-face meeting was held in January 2015 coinciding with the WAO Symposium in Rome, Italy. During the meeting the guideline panel discussed specific questions, the existing research evidence and made recommendations.

### Disclosure of potential conflicts of interest

Guideline panel members disclosed all potential conflicts of interest according to the World Health Organization policies. The chairs (AF, RP and HJS) reviewed and resolved all potential conflicts of interest of panel members (see online Additional file 1 for the list of declared conflicts of interest for all panel members). During all deliberations, panel members with potential conflicts of interest recused themselves from final decisions about recommendations.

The WAO provided meeting facilities during its Symposium and financial support to perform systematic reviews. The views and interests of the WAO as well as of any commercial entity that provided external funding for WAO had no influence on the final recommendations.

### Formulating specific clinical questions and determining outcomes of interest

We used the GRADEpro Guideline Development Tool (www.gradepro.org) [11] and SurveyMonkey (surveymonkey.com) to brainstorm and subsequently prioritize questions related to the use of prebiotics for the prevention of allergy.

The following questions were chosen to be addressed in this document:

1. Should prebiotics vs. no prebiotics be used in pregnant women?
2. Should prebiotics vs. no prebiotics be used in breastfeeding women?
3. Should prebiotics vs. no prebiotics be used in infants?

The guideline panel selected outcomes of interest for each question following the general approach suggested by the GRADE Working Group [1]. All outcomes were identified a priori and the panel explicitly rated their relative importance for decision-making using an online software. Rating outcomes by their relative importance can help focus attention on those outcomes that are considered most important and help to resolve or clarify potential disagreements.

### Evidence review and development of clinical recommendations

Evidence summaries for each question were prepared by the methodologists (JB, CCG, JJYN, GPM, YZ, and HJS) following the GRADE approach and using the Guideline Development Tool (www.gradepro.org). All guideline panel members reviewed the summaries of evidence and made corrections when appropriate. We based the evidence summaries on a systematic review of the literature performed specifically for these guidelines ***(Reference****. in preparation*). An updated search strategy (presented in the online Additional file 2) was performed on July 29, 2015 that did not provide any additional studies. We followed the methods outlined in the Cochrane Handbook for Systematic Reviews of Interventions (handbook.cochrane.org) and assessed the risk of bias at the outcome level using the Cochrane Collaboration’s risk of bias tool [12]. Subsequently, we assessed the quality of the body of evidence (also known as certainty of evidence or confidence in the estimated effects) for each of the outcomes of interest following the GRADE approach based on the following criteria: risk of bias, precision, consistency and magnitude of the estimates of effects, directness of the evidence, risk of publications bias, presence of dose–effect relationship, and an assessment of the effect of residual, opposing confounding. Quality (also called certainty) was categorized into 4 levels ranging from very low to high. In addition, we searched for evidence about values and preferences and cost of prebiotic supplementation. We prepared the evidence-to-decision tables based on the estimates of the health effects, values and preferences and resource use.

During the meeting the guideline panel developed recommendations based on the evidence summaries and the evidence-to-decision frameworks [13–15]. For each recommendation, the guideline panel considered and agreed on the following: the quality of the evidence, the health balance of benefits and harms of the compared management options and the assumptions about the values and preferences associated with the decision. The guideline panel also explicitly took into account the possible extent of resource use associated with alternative management options, feasibility, acceptability and equity considerations. Recommendations and their strength were decided by consensus and no recommendation required voting based on the balance of all desirable and undesirable consequences. The panel agreed on the final wording of recommendations and remarks with further qualifications for each recommendation. The final document including recommendations was reviewed and approved by all members of the guideline panel.

We labelled the recommendations as either “strong” or “conditional” according to the GRADE approach. We used the words “the panel members recommend” for strong recommendations and “suggest” for conditional recommendations. Table 1 provides the suggested interpretation of strong and conditional recommendations by patients, clinicians and health care policy makers.

**Table 1:** Interpretation of strong and conditional recommendations

Implications for:	Strong recommendation	Conditional recommendation
Patients	Most individuals in this situation would want the recommended course of action, and only a small proportion would not.	The majority of individuals in this situation would want the suggested course of action, but many would not.
Clinicians	Most individuals should receive the intervention. Adherence to this recommendation according to the guideline could be used as a quality criterion or performance indicator. Formal decision aids are not likely to be needed to help individuals make decisions consistent with their values and preferences.	Recognize that different choices will be appropriate for individual patients and that you must help each patient arrive at a management decision consistent with his or her values and preferences. Decision aids may be useful in helping individuals to make decisions consistent with their values and preferences.
Policy makers	The recommendation can be adopted as policy in most situations.	Policymaking will require substantial debate and involvement of various stakeholders.

### Document review

A final draft document was reviewed by each member of the guideline panel, finalized, approved, and submitted to the WAO for peer review. The document was revised to incorporate the pertinent comments suggested by the external reviewers.

### How to use these guidelines

The WAO GLAD-P guidelines about the use of prebiotics in the prevention of allergy in children are not intended to impose a standard of care. They provide the basis for rational decisions. Clinicians, patients, third-party payers, institutional review committees, other stakeholders, or the courts should never view these recommendations as dictates. No recommendation can take into account all of the often-compelling unique individual circumstances. Therefore, no one charged with evaluating health care professionals’ actions should attempt to apply the recommendations from these guidelines by rote or in a blanket fashion.

Statements about the underlying values and preferences, qualifying remarks and the evidence to decision frameworks accompanying each recommendation are its integral parts and serve to facilitate more accurate interpretation. They should never be omitted when quoting or translating recommendations from these guidelines.

## Recommendations

### Question 1. Should prebiotics versus no prebiotics be used in pregnant women?

#### Summary of the evidence

We found no systematic reviews, individual randomised trials or observational studies that addressed this question.

#### Conclusions and research needs

The guideline panel decided that under the current circumstances, they were not able to make an informed judgment about the balance of potential desirable and undesirable consequences of using prebiotic supplements in pregnant women. The panel determined that there is a need for well designed and executed randomized trial of prebiotic supplementation in pregnant women that would measure development of allergy in their children as well as other outcomes important in this context. The panel identified the following additional research questions: 1) is the effect of natural prebiotics in food different from that of supplements, 2) is there an added benefit from prebiotic supplementation on top of natural prebiotics, and 3) is there a differential effect between the different types of prebiotics.

#### What others are saying

We found no other guidelines or position statements that made specific recommendations about the use of prebiotics in pregnant women.

#### Recommendation 1

The WAO guideline panel chose to provide no recommendation at this time owing to lack of experimental and observational studies in which prebiotic supplements would be used in pregnant women.

### Question 2. Should prebiotics versus no prebiotics be used in breastfeeding mothers?

#### Summary of the evidence

We found no systematic reviews or individual randomised trials or observational studies that addressed this question.

#### Conclusions and research needs

Similarly to Question 1, the guideline panel decided that under current circumstances they were not able to make an informed judgment about the balance of potential desirable and undesirable consequences of using prebiotic supplements in breastfeeding mothers. The panel determined that there is a need for randomized trials of prebiotics in breastfeeding mothers. The additional questions provided in the discussion of Question 1 related to the effects of natural prebiotics vs. their supplements are equally relevant in the context of Question 2.

#### What others are saying

We found no other guidelines or position statements that would make specific recommendations about the use of prebiotics in breastfeeding mothers.

#### Recommendation 2

The WAO guideline panel chose to provide no recommendation at this time owing to lack of experimental and observational studies in which prebiotic supplements would be used in breastfeeding mothers.

### Question 3. Should prebiotics versus no prebiotics be used in healthy infants?

#### Summary of the evidence

We found three systematic reviews [16–18] that addressed this question but only the review by Osborn [18] assessed the use of prebiotics specifically for the prevention of allergy in infants. We identified 15 additional studies [19–33] published after the search for the Osborn review was completed and studies that measured other outcomes relevant in this context (e.g., adverse effects of prebiotic supplementation). Altogether, there were 18 randomized trials [19–36] that addressed this question. All studies included infants (from term newborns to 3 years of age) not being breastfed. Prebiotics were either added to a cereal (two studies) [24, 28], used as an oral capsule (one study), [29] or in milk formula (15 studies) [19–23, 25–27, 30–36]. Online Additional file 3 presents the characteristics of all included studies.

Five studies reported the effect of prebiotic supplementation on development of eczema (either specifically atopic eczema with positive skin tests, or any eczema), [19, 25, 26, 34, 35] two studies reported development of asthma or recurrent wheezing, [19, 25] one study [25] assessed the risk of developing food allergy, and twelve studies [20–24, 27, 29–31, 33, 34, 36] measured nutritional status. No studies reported the risk of developing allergic rhinitis or the composite outcome of “any allergy”.

#### Benefits

Prebiotic supplemented during the first year of life reduced the risk of developing asthma or recurrent wheezing (RR: 0.37, 95 % CI: 0.17 to 0.80) and the risk of developing food allergy (RR: 0.28, 95 % CI: 0.08 to 1.00). Prebiotics also probably reduce the risk for developing eczema in infants but the estimate is imprecise and confidence interval does not exclude no effect (RR: 0.57, 95 % CI: 0.30 to 1.08). Studies assessing nutritional status assessed as weight gain found no difference between those receiving prebiotics and control groups (standardized mean difference [SMD]: 0.06, 95 % CI: -0.02 to 0.15). Overall the certainty of these estimated effects is very low owing to the serious risk of bias in the studies and imprecision of the estimates (see evidence profile in the online Additional file 4).

#### Harms and burden

Seven studies reported adverse effects [20, 22, 23, 28, 30, 32, 34] with a median risk of 34 % in the control groups. All adverse effects were judged to be minor. The risk of adverse effects was not different among those receiving prebiotics and those receiving placebo (RR: 1.03, 95 % CI: 0.93 to 1.14). The most commonly reported adverse events were: rash, gastro-esophageal reflux, emesis, diarrhea, cramps, crying or “colic”. These events did not lead to withdrawal from the studies. There is low certainty in the estimate of the risk of adverse effects due to inadequate reporting in primary studies. However, given the available evidence, the guideline panel considered the risk of adverse effects most likely to be low.

#### Other decision criteria and considerations

Formulas supplemented with prebiotics should not be considered a substitute for breast milk. All current evidence concerns infants who were not breastfed, thus, the availability of such formulas should not influence the decision to breastfeed and/or the duration of breastfeeding.

#### Conclusions and research needs

The guideline panel determined that there is a low certainty of a net benefit from using prebiotics in infants. Based on the body of available evidence, it is likely that prebiotic supplementation in infants reduces the risk of developing recurrent wheezing and possibly also the development of food allergy. There is very low certainty that there is an effect of prebiotics on other outcomes. However, because of low certainty of evidence or no published information about other outcomes, the fact that we did not find the evidence of an effect on these outcomes does not imply that such an effect does not exist.

The panel identified the following additional research questions: 1) is the effect of natural prebiotics in food different from that of supplementation, 2) is there an added benefit from prebiotic supplementation in addition to natural prebiotics, and 3) is there a differential effect between the different types of prebiotics. Future research from rigorously designed and well-executed randomized trials may have an important impact on this recommendation.

#### What others are saying

The European Academy of Allergy and Clinical Immunology (EAACI) Food Allergy and Anaphylaxis Guidelines state that “there is no evidence to recommend prebiotics or probiotics or other dietary supplements based on particular nutrients to prevent food allergy.”[37]. The European Society for Pediatric Gastroenterology, Hepatology, and Nutrition (ESPGHAN) and North American Society for Pediatric Gastroenterology, Hepatology, and Nutrition (NASPGHAN) Committee on Nutrition consider that although some benefits might be conferred to the administration of prebiotics in infant formulas, these results should not influence practice until confirmed by additional studies. They do not recommend routine administration of prebiotics for the prevention of allergies [38]. A guideline from the Scottish Intercollegiate Guidelines Network (SIGN) for the management of atopic eczema states that it is not possible to make recommendations for prebiotics to prevent allergy based on the current evidence [39].

#### Recommendation 3

The WAO guideline panel suggests prebiotic supplementation in not-exclusively breastfed infants, both at high and at low risk for developing allergy (conditional recommendation, low certainty of evidence).

The WAO guideline panel suggests that clinicians and parents do not use prebiotic supplementation in exclusively breastfed infants (conditional recommendation, low certainty of the evidence).

#### Values and preferences

The recommendation to use prebiotics in not-exclusively breastfed infants places a relatively higher value on possible prevention of allergies and a relatively lower value on additional cost of prebiotic supplementation. The recommendation to not use prebiotics in exclusively breastfed infants places a relatively higher value on avoiding additional cost and burden of implementation of such supplementation and a relatively lower value on uncertain effect on prevention of allergies (currently there are also no studies of health effects of prebiotics in exclusively breastfed infants and the panel found it not justified to extrapolate from the effects observed in non-exclusively breastfed infants).

#### Explanations and other considerations

These recommendations should not lead to any change in the practice or promotion of breastfeeding of infants. Formulas supplemented with prebiotics should not be considered a substitute for breast milk. Sole availability of such formulas should not influence the decision to breastfeed and/or the duration of breastfeeding. These recommendations apply to otherwise healthy infants in whom prebiotics would be used with the goal of preventing allergies. See the evidence to recommendation table in online Additional file 5.

## Priorities for revision of the guidelines

### Plans for updating these guidelines

Guidelines are living documents. To remain useful, they need to be updated regularly as new information accumulates. A revision of this document will be needed, because there was limited evidence for many clinical questions. This document will be updated when major new research is published. The need for update will be determined not later than in 2019.

### Updating or adapting recommendations locally

The methods used to develop these guidelines are transparent. The recommendations have been developed to be as specific and detailed as possible without losing sight of the simplicity of the document. Since GLAD-P are meant as international guidelines, the guideline panel encourages feedback on all its aspects including their applicability in individual countries. This feedback will be considered when revising the document.

Adaptation of these guidelines will be necessary in many circumstances. Depending on when such a process takes place, the following steps should be taken:

- ▪ Appointing a guideline committee comprising clinicians and methodologists
- ▪ Determining the scope of the local guidelines
- ▪ Defining the relevant clinical questions to be addressed in local guidelines
- ▪ Reviewing and updating the evidence profiles and evidence-to-decision frameworks if necessary
- ▪ Reviewing the recommendations in the GLAD-P guidelines (the recommendations may need to be modified at a local level, depending on the local values and preferences, availability of medications, costs, etc.)
- ▪ Disseminating the guidelines, with a clear “use by” date
- ▪ Developing a method to obtain feedback and plans for review and update.

## Priorities for research

During the guideline development process we identified a need for more data on specific topics. This resulted in the following recommendations for research. We summarize these gaps in the evidence as research recommendations, to assist those in a position to provide such information through the design and execution of specific research projects.

Specific research needs to be addressed:

1. Development of instruments for evaluating the risk of allergy in children (the family history predicts only about 30 % of the population risk).
2. Evaluation of effects of using prebiotics in breastfeeding mothers.
3. Evaluation of effects of using prebiotics in pregnant women.
4. Evaluation of the effects of different ways of administration of prebiotics, e.g. as milk hydrolysed formula, dairy supplements, stand-alone supplements, etc.
5. Performance of rigorously designed, adequately powered, and well-executed randomized trials of the different prebiotics (e.g., GOS vs. FOS) with well-defined patient important outcomes.
6. The impact of sub-groups in the effect (if any) of prebiotics on the prevention of allergies, such as:
  - Infants at high vs. low risk of allergies
  - Those being exclusively breastfed vs. not exclusively breastfed
  - Those born by caesarean-section vs. those born by vaginal delivery
7. Large observational studies might be the only design feasible for detecting rare adverse events

## Additional files

Additional file 1:
**Conflict of interest declaration.** (DOCX 506 kb) Additional file 2:
**Search strategies.** (PDF 33 kb) Additional file 3:
**Included studies.** (PDF 140 kb) Additional file 4:
**Evidence profiles.** (DOCX 131 kb) Additional file 5:
**Evidence to Decision Framework.** (DOCX 163 kb)
