# Supplementary material for: World Allergy Organization-McMaster University Guidelines for Allergic Disease Prevention (GLAD-P): Prebiotics
Source: World Allergy Organ J. 2016 Mar 1;9:10. doi: 10.1186/s40413-016-0102-7 (PMC4772464; doi:10.1186/s40413-016-0102-7)
Supplement: Additional file 1: — Conflict of interest declaration. (DOCX 506 kb) [file 40413_2016_102_MOESM1_ESM.docx]

## The following is the declaration of potential conflicts of interest (within last 4 years) for the guideline panel members and authors of this work

| Panel member | Declaration |
| --- | --- |
| Kangmo Ahn | Declares no conflicts of interest related to this guideline |
| Suleiman Al-Hammadi | Received honoraria for speakers bureau, giving talks, and sponsorship at meetings from Danone Nutrition. |
| Kirsten Beyer | Consult as technical advisor for DuPont, Unilever and Danone. |
| Jan Brożek | Received research support from WAO for development of DRACMA and GLAD-P guidelines |
| Wesley Burks (not present) | Current consulting agreements with: Dynavax Technologies Corp., Genalyte, GLG Research, Perrigo Company, Regeneron Pharmaceuticals, NIH Grant support c. Abbott Laboratories, Levine's Children's Hospital, Mylan Speciality, Perosphere, Inc. Past consulting agreements: ActoGeniX, Curalogic, Dow AgroSciences, ExploraMed Development, McNeill Nutritionals, Merck, Novartis Pharma AG, Sanofi-Aventis US, Schering Plough, Unilver |
| Giorgio Walter Canonica (not present) | Declares no conflicts of interest related to this guideline |
| Carlos Cuello-García | Declares no conflicts of interest related to this guideline. Part of the scholarship is supported by WAO |
| Motohiro Ebisawa (not present) | Declares no conflicts of interest related to this guideline |
| Alessandro Fiocchi | Research support from Danone. Has received travel support for meetings for presentation of DRACMA guidelines in South East Asia from Danone Malaysia. Support for travel and honorarium for speaking from Ordesa Group. |
| Rose Kamenwa | Declares no conflicts of interest related to this guideline |
| Bee Wah Lee (not present) | Received research support from Abbott Nutrition, Danone Nutrition, Nestle Nutrition Honoraria for speakers bureau, giving talks, sponsorship at meetings |
| Haiqi Li (not present) | Declares no conflicts of interest related to this guideline |
| Ruby Pawankar | Received support from Danone Asia Pacific for act as a speaker at a Food allergy Asia Pacific symposium 2012 |
| Susan Prescott | Advisory Board - Nestle Nutrition Institute (Australasia), Advisory Board for Danone (Asia Pacific), investigator initiated trial for which Danone provided prebiotic. |
| John Riva | Declares no conflicts of interest related to this guideline |
| Lanny Rosenwasser | Research Grant from Novartis /Genentech Roche (finished in 2011). Advisory board and speakers board for Astra Zeneca and Novartis/Genentech (last applicable in 2012), 5 patents on the biology of IL-1 and it's uses. |
| Hugh Sampson | Consultant– Danone Research for Specialized Nutrition Scientific Advisory Committee. |
| Holger Schünemann | Received research support from WAO for development of DRACMA and GLAD-P guidelines |
| Michael D. Spigler (not here) | Declares no conflicts of interest related to this guideline |
| Luigi Terracciano (not here) | Heinz-Plada Italy Medical consultant for website. Travel support from World Allergy Organization. |
| Andrea Vereda-Ortiz | Declares no conflicts of interest related to this guideline |
| Susan Waserman | Declares no conflicts of interest related to this guideline |
| Juan José Yepes Nuñez | Declares no conflicts of interest related to this guideline. Part of scholarship paid by WAO funding received by HJS and JLB for developing these guidelines |
| Yuan Zhang | Declares no conflicts of interest related to this guideline |
| Gian Paolo Morgano | Declares no conflicts of interest related to this guideline |
| Arnav Agarwal | Declares no conflicts of interest related to this guideline |
| Shreyas Ghandi | Declares no conflicts of interest related to this guideline |

WAO supported travel for all panel members to attend the Rome meeting
