# Supplementary material for: World Allergy Organization-McMaster University Guidelines for Allergic Disease Prevention (GLAD-P): Prebiotics
Source: World Allergy Organ J. 2016 Mar 1;9:10. doi: 10.1186/s40413-016-0102-7 (PMC4772464; doi:10.1186/s40413-016-0102-7)
Supplement: Additional file 2: — Search strategies. (PDF 33 kb) [file 40413_2016_102_MOESM2_ESM.pdf]

SEARCH STRATEGIES - PREBIOTICS

#	MEDLINE OVID
1	randomized controlled trial.pt.
2	controlled clinical trial.pt.
3	randomized.ab.
4	placebo.ab.
5	clinical trials as topic.sh.
6	randomly.ab.
7	trial.ti.
8	1 OR 2 OR 3 OR 4 OR 5 OR 6 OR 7
9	exp animals/ NOT humans.sh.
10	8 NOT 9
11	exp prebiotics/
12	oligosaccharide$.ab,ti,tw.
13	fructo-oligosaccharide$.ab,ti,tw.
14	galacto-oligosaccharide$.ab,ti,tw.
15	inulin.ab,ti,tw.
16	lactulose.ab,ti,tw.
17	11 OR 12 OR 13 OR 14 OR 15 OR 16
18	infant$.ab,ti,tw.
19	newborn.ab,ti,tw.
20	pediatric.ab,ti,tw.
21	paediatric.ab,ti,tw.
22	child$.ab,ti,tw.
23	exp pregnancy/
24	exp breast feeding/
25	18 OR 19 OR 20 OR 21 OR 22 OR 23 OR 24
26	10 AND 17 AND 25

#	EMBASE OVID
1	random:.tw
2	placebo:.mp
3	double-blind:.tw
4	1 OR 2 OR 3
5	exp animals/ NOT humans.sh.
6	4 NOT 5
7	exp prebiotics/
8	oligosaccharide$.ab,ti,tw.
9	fructo-oligosaccharide$.ab,ti,tw.
10	galacto-oligosaccharide$.ab,ti,tw.
11	inulin.ab,ti,tw.
12	lactulose.ab,ti,tw.
13	7 OR 8 OR 9 OR 10 OR 11 OR 12
14	infant$.ab,ti,tw.
15	newborn.ab,ti,tw.
16	pediatric.ab,ti,tw.
17	paediatric.ab,ti,tw.
18	child$.ab,ti,tw.
19	exp pregnancy/
20	exp breast feeding/
21	14 OR 15 OR 16 OR 17 OR 18 OR 19 OR 20
22	6 AND 13 AND 21

#	CENTRAL
1	PREBIOTICS
2	oligosaccharides
3	fructo-oligosaccharides
4	galacto-oligosaccharides
5	inulin
6	lactulose
7	1 OR 2 OR 3 OR 4 OR 5 OR 6
8	infant?
9	newborn?
10	pediatric?
11	paediatric?
12	child?
13	PREGNANCY
14	BREAST FEEDING
15	8 OR 9 OR 10 OR 11 OR 12 OR 13 OR 14
16	7 AND 15

The search strategies were performed on 29 January 2015, and repeated on 29 July 2015.
