# Supplementary material for: World Allergy Organization-McMaster University Guidelines for Allergic Disease Prevention (GLAD-P): Prebiotics
Source: World Allergy Organ J. 2016 Mar 1;9:10. doi: 10.1186/s40413-016-0102-7 (PMC4772464; doi:10.1186/s40413-016-0102-7)
Supplement: Additional file 3: — Included studies. (PDF 140 kb) [file 40413_2016_102_MOESM3_ESM.pdf]

Characteristics of included studies

Study	Age group(s)	Risk of allergies group	Prebiotics	Dosage and frequency	Duration of treatment (weeks)	Comparison	Outcomes assessed
Arslanoglu 2008	Infants	High risk	Oligosaccharides (FOS/GOS) in HA formula	0.8 g /100 mL	24	Formula with 0.8 g maltodextrin/100 ml as placebo	Eczema, asthma
Ashley 2012	Newborns	Average risk	Oligosaccharides (FOS/GOS) in formula	0.4 g/100 mL	15	placebo (maltodextrin) in formula	Adverse events, nutrition
Bruzzese 2009	Newborns and infants	Average risk	Oligosaccharides (FOS/GOS) in formula	0.4 g/100 mL	52	formula without prebiotics	Nutrition
Closa-Monasterolo 2013	Newborns and infants	Can't tell or Unknown	Oligofructose; SYN1 (0.8 g/dl) half oligofructose, half inulin in formula	as formula, ad libitum	16	control formula placebo (maltodextrin)	Adverse events, nutrition
Costalos 2008	Infants	Average risk	Oligosaccharides (FOS/GOS) in formula	0.4 g/100 mL	12	formula without added prebiotics	Adverse events, nutrition
Duggan 2003	Infants	Average risk	oligofructose in cereal	0.55 g of prebiotic per 15 g of cereal	24	cereal without prebiotic	Nutrition
Gruber 2010	Newborns and infants	Average risk	Oligosaccharides (FOS/GOS) in formula	0.8g/100 mL with 6.8g/L neutral and 1.2g/L pAOS	24	Formula without added prebiotics	Eczema
Ivakhnenko 2013	Newborns and infants	Can't tell or Unknown	Oligosaccharides (FOS/GOS) in formula	0.8 g/100 mL	32	formula without FOS/GOS	Eczema

Study	Age group(s)	Risk of allergies group	Prebiotics	Dosage and frequency	Duration of treatment (weeks)	Comparison	Outcomes assessed
Lista 2007	Newborns	High risk	Oligosaccharides (FOS/GOS) in HA formula	not specified	13	Hypoallergenic formula without prebiotics	Eczema, asthma, food allergy
Magne 2008	Infants	Average risk	Oligosaccharides (FOS/GOS) in whey based formula	0.54 g/100mL	21	whey-based formula without prebiotics	Nutrition
Moore 2003	Infants	Can't tell or Unknown	Oligosaccharides (FOS/GOS) in cereal	0·03 g FOS/g cereal (0·75 g FOS/25 g serving of cereal)	4	placebo (0·03 g of maltodextrin per one gram of cereal)	Adverse events
Moro 2002	Infants	Can't tell or Unknown	Oligosaccharides (FOS/GOS) in formula	0.4 g/dl	4	placebo (maltodextrin) in formula	Nutrition
Moro 2006	Infants	Can't tell or Unknown	Oligosaccharides (FOS/GOS) in formula	0.8 g/dl	24	placebo (maltodextrin) in formula	Eczema
Partty 2013	Infants	Can't tell or Unknown	Oligosaccharides (FOS/GOS) in capsule	600 mg/d each day for 30 days, then 600 mg twice a day	52	placebo capsules with cellulose and dextrose	Nutrition
Piemontese 2011	Newborns and infants	Can't tell or Unknown	Oligosaccharides (FOS/GOS) in formula	8 g/L with 6.8 g/L neutral and 1.2 g/L pectin acidic-oligosacchari des	32	formula without prebiotics	Adverse events, nutrition

Study	Age group(s)	Risk of allergies group	Prebiotics	Dosage and frequency	Duration of treatment (weeks)	Comparison	Outcomes assessed
Salvini 2011	Newborns and infants	Can't tell or Unknown	Oligosaccharides (FOS/GOS) in formula	8 g/l	52	Formula without added prebiotics	Nutrition
Scholtens 2008	Newborns and infants	Both	Oligosaccharides (FOS/GOS) in formula	6 g/L scGOS/ lcFOS in a ratio of 9:1	26	Control infant milk formula without prebiotics	Adverse events
Weereman-Wauters 2011	Newborns	Can't tell or Unknown	Oligosaccharides (FOS/GOS) in formula	0.8 g/l. GOS:FOS formula, 90:10 with long-chain inulin	4	Standard formula without prebiotics	Nutrition
Ziegler 2007	Newborns and infants	Can't tell or Unknown	PDX, GOS, and LOS in formula	in formula	17	formula without added prebiotics	Adverse events, nutrition

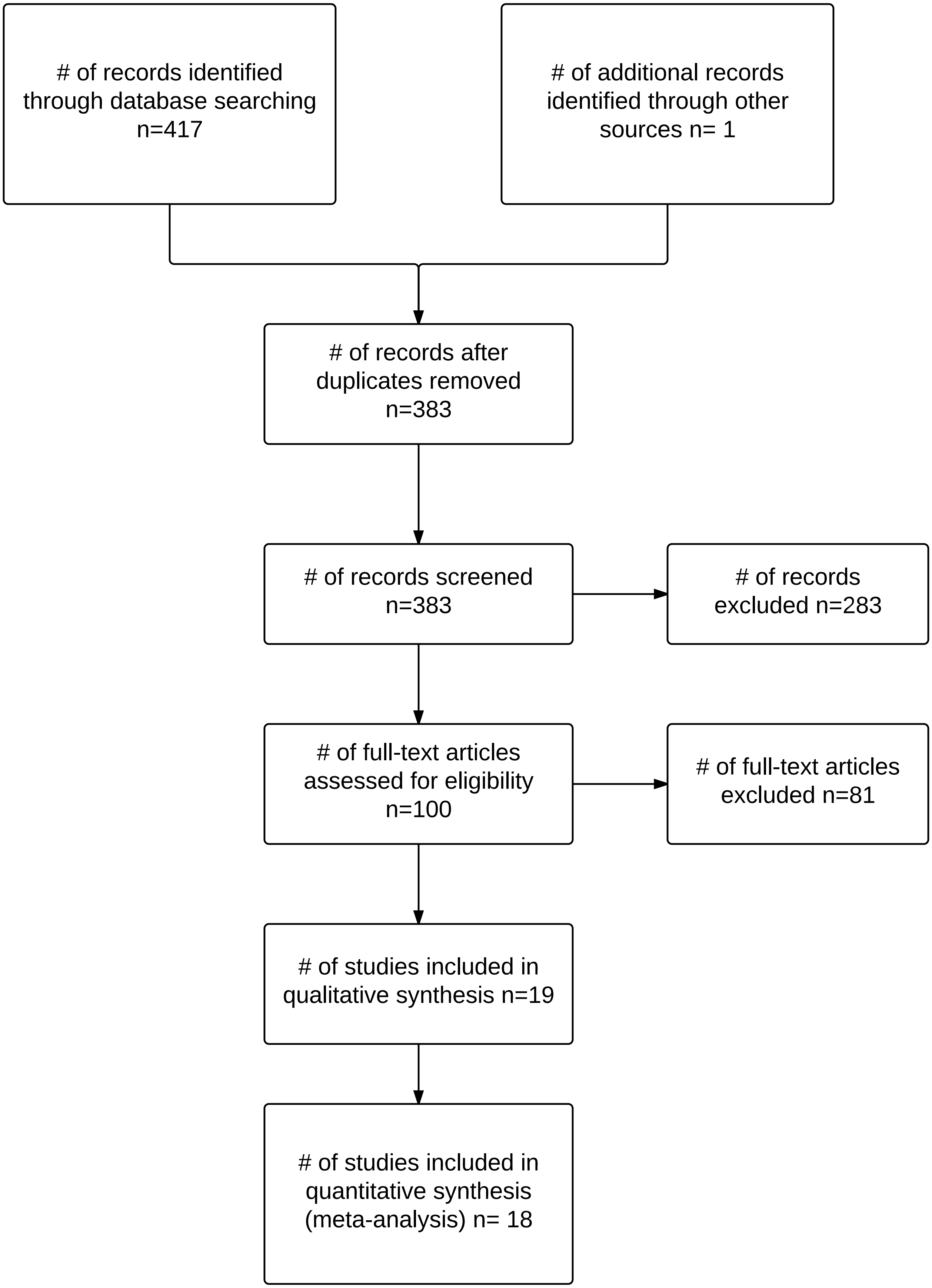
# of records identified through database searching n=417
# of additional records identified through other sources n= 1
# of records after duplicates removed n=383
# of records screened n=383
# of records excluded n=283
# of full-text articles assessed for eligibility n=100
# of full-text articles excluded n=81
# of studies included in qualitative synthesis n=19
# of studies included in quantitative synthesis (meta-analysis) n= 18
