# Supplementary material for: World Allergy Organization-McMaster University Guidelines for Allergic Disease Prevention (GLAD-P): Prebiotics
Source: World Allergy Organ J. 2016 Mar 1;9:10. doi: 10.1186/s40413-016-0102-7 (PMC4772464; doi:10.1186/s40413-016-0102-7)
Supplement: Additional file 4: — Evidence profiles. (DOCX 131 kb) [file 40413_2016_102_MOESM4_ESM.docx]

**Question**: Prebiotics compared to no prebiotics for the prevention of allergies

**Settings**: ambulatory care

**Author(s)**: Carlos Cuello, Juan J. Yepes, Yuan Zhang, Gian Paolo Morgano, Jan Brożek, Holger Schünemann

**Date**: January 2015

**Bibliography (systematic reviews)**:  Cuello-Garcia, et al. 2015 (in process).

| **Quality assessment** | | | | | | | **№ of patients** | | **Effect** | | **Quality** | **Importance** |
| --- | --- | --- | --- | --- | --- | --- | --- | --- | --- | --- | --- | --- |
| **№ of studies** | **Study design** | **Risk of bias** | **Inconsistency** | **Indirectness** | **Imprecision** | **Other considerations** | **prebiotics** | **no prebiotics** | **Relative**  **(95% CI)** | **Absolute**  **(95% CI)** |  |  |
| **Development of eczema** (any eczema) (follow up: range 3 to 24 months) | | | | | | | | | | | | |
| 5 | randomised trials | serious ^1^ | not serious | not serious | serious ^2^ | none | 47/683 (6.9%) | 75/630 (11.9%) | **RR 0.57**  (0.3 to 1.08) | 51 fewer per 1000  (from 83 fewer to 10 more) | ⨁⨁◯◯  LOW | CRITICAL |
| **Development of allergic rhinitis** | | | | | | | | | | | | |
| 0 | – | – | – | – | – | – | – | – | – | – | – | CRITICAL |
| **Development of asthma and/or recurrent wheezing** (follow up: range 18 to 24 months) | | | | | | | | | | | | |
| 2 | randomised trials | serious ^3^ | not serious | serious^11^ | serious ^4^ | none | 8/128 (6.3%) | 21/121 (17.4%) | **RR 0.37**  (0.17 to 0.8) | 109 fewer per 1000  (from 35 fewer to 144 fewer) | ⨁◯◯◯  VERY LOW | CRITICAL |
| **Development of food allergy** (follow up: median 18 months) | | | | | | | | | | | | |
| 1 | randomised trials | serious ^5^ | not serious | very serious^10^ | serious ^2,6^ | none | 3/62 (4.8%) | 9/53 (17.0%) | **RR 0.28**  (0.08 to 1.00) | 122 fewer per 1000  (from 0 fewer to 156 fewer) | ⨁◯◯◯  VERY LOW | CRITICAL |
| **Development of any allergy** | | | | | | | | | | | | |
| 0 | – | – | – | – | – | – | – | – | – | – | – | IMPORTANT |
| **Adverse events** (follow up: range 1 to 12 months) | | | | | | | | | | | | |
| 7 | randomised trials | serious ^7^ | not serious | not serious | serious ^8^ | none | 350/970 (36.1%) | 329/948 (34.7%) | **RR 1.03**  (0.93 to 1.14) | 21 more per 1000  (from 17 fewer to 66 more) | ⨁⨁◯◯  LOW | CRITICAL |
| **Nutritional status** (follow up: range 1 to 12 months; assessed with: mean weight gained per day or the final weight assessed by clinicians; better indicated by higher values) | | | | | | | | | | | | |
| 12 | randomised trials | serious ^9^ | not serious | not serious | not serious | none | 1162 | 1136 | - | SMD **0.06 higher**  (0.02 lower to 0.15 higher) | ⨁⨁⨁◯  MODERATE | CRITICAL |

SMD – standardised mean difference, RR – relative risk

1. Random sequence generation was unclear in 4 studies and in 2 studies 20% to 30% of participants were lost to follow-up
2. 95% CI does not exclude an appreciable clinical benefit or no difference.
3. 20% and 30% of patients lost to follow-up in both studies; there is a concern about blinded assessment of outcomes and inadequate description of the allocation concealment
4. Only 29 events.
5. Only one study with 30% lost to follow-up and inadequate description of blind outcome assessment
6. Only 12 events.
7. There was 20 to 30% lost to follow-up in 4 studies; 5 studies did not describe blinded outcome assessment, and random sequence generation and allocation concealment were not adequately described (in 2 and 3 studies, respectively).
8. 95% confidence interval does not exclude an appreciable harm
9. Blind outcome assessment was not described in 8 studies and in 2 studies there was large number of patients lost to follow-up
10. There is a concern that only one study reported this outcome and did not describe how the outcome was measured (it seems to be a self-reported adverse reaction to food); there is very serious concern that this outcome does not reflect allergic reactions at all.
11. Development of asthma was not directly measured and the guideline panel was uncertain whether recurrent wheezing is an appropriate proxy for development of asthma
