# Supplementary material for: World Allergy Organization-McMaster University Guidelines for Allergic Disease Prevention (GLAD-P): Prebiotics
Source: World Allergy Organ J. 2016 Mar 1;9:10. doi: 10.1186/s40413-016-0102-7 (PMC4772464; doi:10.1186/s40413-016-0102-7)
Supplement: Additional file 5: — Evidence to Decision Framework. (DOCX 163 kb) [file 40413_2016_102_MOESM5_ESM.docx]

| **Should pr*e*biotics be used in infants for the prevention of allergy?** | |
| --- | --- |
| ***Population:*** healthy infants  ***Intervention:*** prebiotic supplementation  ***Comparison:*** no prebiotic supplementation  ***Main outcomes:*** development of allergy, nutritional status, adverse effects  ***Setting:*** outpatient  ***Perspective:*** population | ***Background:*** Allergic diseases represent a spectrum of health conditions and a worldwide burden in different populations. In infants, its prevalence depends highly on the allergic status of their parents, being approximately of 10% in those without an allergic parent or sibling, versus 20% to 30% in those with an atopic background in their first-degree relatives. Allergic conditions (asthma, rhinitis, eczema, food allergy) are considered an economic burden that can also affects the quality of life of affected populations.  An altered microbial exposure in the gastrointestinal tract is considered to be partly responsible for the increase of allergic diseases. Prebiotics are non-digestible food components that may benefit the host by selectively stimulating the growth or activity of bacteria in the colon, and may decrease the risk for allergies by promoting a balanced growth of the intestinal microbiota. |

|  | **Criteria** | **Judgements** | **Research evidence** | **Additional considerations** |
| --- | --- | --- | --- | --- |
| Problem | **Is there a problem priority?** | ○ No ○ Probably no ○ Uncertain ○ Probably yes **● Yes** ○ Varies | Baseline risks for development of allergy in children are high. In the included studies it was in the range of 12% to 17%. |  |
| Benefits & harms of the options | **What is the overall certainty of this evidence?** | ○ No included studies **● Very low** ○ Low ○ Moderate ○ High | **Values and preferences**   \| **Outcome** \| **Value placed on an outcome (utility)^1^** \| **Certainty of evidence** \| \| --- \| --- \| --- \| \| **Eczema^2^** \|  \| VERY LOW \| \| Well controlled \| 0.98 \| \| Mild \| 0.86 \| \| Moderate \| 0.69 \| \| Severe \| – \| \| **Allergic rhinitis^3^** \|  \| VERY LOW \| \| No current symptoms \| 0.78 \| \| Mild \| 0.76 \| \| Moderate to severe \| 0.74 \| \| Severe rhinitis & mild asthma \| 0.73 \| \| Severe rhinitis and moderate to severe asthma \| 0.70 \| \| **Asthma or recurrent wheezing**^4,5^ \|  \| VERY LOW \| \| overall \| 0.66 to 0.96 (range) \| \| Mild \| 0.55 to 0.79 (range) \| \| Moderate \| 0.49 to 0.7 (range) \| \| Severe \| 0.16 to 0.28 (range) \| \| **Food allergy^2^** \| 0.9 \| VERY LOW \| \| **Adverse events** \| – \| – \| \| **Nutritional status** \| – \| – \| \| **Any allergy** \| – \| – \|   1 Utility is expressed on a scale from 0 (death) to 1 (perfect health)  2 measured with HUI  3 no information how utilities were measured; values for a cost-utility analysis  4 measured with multiple instruments  5 Utilities derived for both adults and children  **Summary of findings**   \| Outcome \| Without prebiotics  (per 1000) \| With prebiotics  (per 1000) \| **Difference**  **(95% CI)**  (per 1000) \| **Relative effect**  **(95% CI)** \| **Certainty of the evidence**  **(GRADE)** \| \| --- \| --- \| --- \| --- \| --- \| --- \| \| Eczema (general) \| 119 \| **68**  (36 to 129) \| 51 fewer  (10 to 83 fewer) \| **RR 0.57** (0.3 to 1.08) \| LOW \| \| Allergic rhinitis \| ­– \| – \| – \| – \| – \| \| Asthma / recurrent wheezing \| 174 \| **64**  (30 to 139) \| 109 fewer  (35 to 144 fewer) \| **RR 0.37** (0.17 to 0.80) \| VERY LOW \| \| Food allergy \| 170 \| **48**  (14 to 170) \| 122 fewer  (0 to 156 fewer) \| **RR 0.28** (0.08 to 1.00) \| VERY LOW \| \| Adverse events \| 347 per 1000 \| **368 per 1000 (330 to 413)** \| 21 more per 1000 (from 17 fewer to 66 more) \| **RR 1.03** (0.93 to 1.14) \| LOW \| \| Nutritional status \| – \| – \| SMD **0.06 higher** (0.02 lower to 0.15 higher) \| - \| MODERATE \| \| Any allergy \| – \| – \| – \| – \| – \| | The concomitant presence of allergic rhinitis and asthma disrupted the ability to get a good night's sleep (79%), to participate in leisure and sports activities (75%), to concentrate at work or school (69% of adults, 73% of children), and to enjoy social activities (57% of adults, 51% of children). Due to these diseases, many (56%) patients avoided outdoor activities during the allergy season because of worsening asthma symptoms.  Qualitative studies revealed the parental experience and patient experience related to children/teenagers with food allergy. The experiences related to food allergy include: experience of isolation or exclusion or feeling of being constrained, living with risks, adjustment due to disease for both parents and children; emotional tension between family members, frustration and difficulties due to untimely diagnosis and recommendations for food allergy for parents; as well as difficulties to try new food, perceiving parental control as protective for children/teenagers.  Adverse events were minor in all studies and not significantly different between the prebiotic and the placebo arms. Amongst the most common cited adverse events were: Rash, gastroesophageal reflux, emesis, diarrhoea, cramps, crying or "colic". These events did not preclude patients from participating in the studies. Furthermore, if nutritional status is considered as an indirect outcome effect of possible adverse events from the administration of prebiotics, there was no effect on infants' gain weight in the final estimate.  There are case reports about severe allergic reactions to prebiotics (refer JACI paper) |
|  | **Is there important uncertainty about how much people value the main outcomes?** | ○ Important uncertainty or variability **● Possibly important uncertainty and probably no important variability** ○ Probably no important uncertainty or variability ○ No important uncertainty of variability ○ No known undesirable |  |  |
|  | **Are the desirable anticipated effects large?** | ○ No ○ Probably no ○ Uncertain **● Probably yes** ○ Yes ○ Varies |  |  |
|  | **Are the undesirable anticipated effects small?** | ○ No ○ Probably no ○ Uncertain **● Probably yes** ○ Yes ○ Varies |  |  |
|  | **Are the desirable effects large relative to undesirable effects?** | ○ No ○ Probably no ○ Uncertain **● Probably yes** ○ Yes ○ Varies |  |  |
| Resource use | **Are the resources required small?** | ○ No ○ Probably no ○ Uncertain ○ Probably yes ○ Yes **● Varies** |  | Resources required will vary according to the subgroups:  - high risk children, not exclusively breastfed – resources required would be small  - low risk children, not exclusively breastfed – resources required would be larger  - all exclusively breastfed children – resources required probably would not be small |
|  | **Is the incremental cost small relative to the net benefits?** | ○ No ○ Probably no ○ Uncertain ○ Probably yes ○ Yes **● Varies** | A cost-utility analysis of a specific mixture of prebiotics in children at risk of atopic dermatitis (AD) in the Netherlands showed the Incremental Cost-Effectiveness Ratio (ICER) was €472/QALY for prebiotics compared with no prebiotics. This Markov model has taken into account both the short- and long-term consequences of AD. The sensitivity analyses show the ICER was robust and below the threshold of €20,000/QALY under different circumstances. | Resources would probably be small if probiotics were used in children at high risk of eczema and likely 2-5 times higher in low risk children. However, that would likely be still cost effective.  Cost effectiveness is uncertain for exclusively breastfed children. |
| Equity | **What would be the impact on health inequities?** | ○ Increased ○ Probably increased ○ Uncertain **● Probably reduced** ○ Reduced ○ Varies |  | It varies according to the setting. |
| Acceptability | **Is the option acceptable to key stakeholders?** | ○ No ○ Probably no ○ Uncertain ○ Probably yes ○ Yes **● Varies** |  | Based on guideline panel member experience, in North America probably acceptable to all stakeholders, but possibly not acceptable or variable acceptability in other settings. |
| Feasibility | **Is the option feasible to implement?** | ○ No ○ Probably no ○ Uncertain ○ Probably yes ○ Yes **● Varies** |  | Yes, in not exclusively breastfed children. In exclusively breastfed children – probably no. |

| **Recommendation**  **Question: Should prebiotics vs. no prebiotics be used for prevention of allergies?** | | | | | |
| --- | --- | --- | --- | --- | --- |
| **Balance of consequences** | Undesirable consequences *clearly outweigh* desirable consequences in most settings | Undesirable consequences *probably outweigh* desirable consequences in most settings | The balance between desirable and undesirable consequences *is closely balanced or uncertain* | Desirable consequences *probably outweigh* undesirable consequences in most settings | Desirable consequences *clearly outweigh* undesirable consequences in most settings |
|  | ○ | ○ | ○ | **●** | ○ |

| **Type of recommendation** | We recommend against offering this option | We suggest not offering this option | We suggest offering this option | We recommend offering this option |
| --- | --- | --- | --- | --- |
|  | ○ | ○ | **●** | ○ |
| **Recommendation** | The WAO guideline panel suggests using **prebiotic supplementation in non-exclusively breastfed infants**, both at high and low risk for developing allergy (conditional recommendation, very low certainty of the evidence).  The WAO guideline panel suggests not using **prebiotic supplementation in exclusively breastfed infants** (conditional recommendation, very low certainty of the evidence). | | | |
| **Justification** | Using probiotics in non-exclusively breastfed children possibly reduces the risk for developing allergy – parents or caregivers who place a higher value on not adding supplements to infant’s diet may choose otherwise. Further research will likely have an important impact on this recommendation. | | | |
| **Subgroup considerations** | The panel felt uncomfortable to extrapolate the evidence and recommendations to the exclusively breastfed infants because there is no information suggesting the effect of prebiotics in this population, and the effect could be different than that observed in infants that were not breastfed. | | | |
| **Implementation considerations** | It is important that these recommendations should not in any way lead to any change in practice of breastfeeding. | | | |
| **Monitoring and evaluation** | Not applicable | | | |
| **Research possibilities** | We found no evidence on the prebiotic supplementation of pregnant women, breastfeeding mothers or exclusively breastfed infants. More research is needed in these populations. | | | |

You can find an online version of this evidence-to-recommendation framework and evidence profile in the following link:

[http://dbep.gradepro.org/profile/93CF0D1F-6DED-48FD-BFDA-9CE1C028D100](http://dbep.gradepro.org/profile/93CF0D1F-6DED-48FD-BFDA-9CE1C028D100)
